# Brief Engagement and Acceptance Coaching for Hospice Settings (the BEACHeS study): results from a Phase I study of acceptability and initial effectiveness in people with non-curative cancer

**DOI:** 10.1186/s12904-021-00801-7

**Published:** 2021-06-25

**Authors:** Nicholas J. Hulbert-Williams, Sabrina F. Norwood, David Gillanders, Anne M. Finucane, Juliet Spiller, Jenny Strachan, Susan Millington, Joseph Kreft, Brooke Swash

**Affiliations:** 1 Centre for Contextual Behavioural Science, School of Psychology, University of Chester, Chester, UK; 2 School of Health in Social Science, The University of Edinburgh, Edinburgh, UK; 3 Marie Curie Hospice, Edinburgh, UK; 4 Chester, UK

**Keywords:** Acceptance and commitment therapy, Coaching, Quality of life, Distress, Palliative, Cancer, Hospice

## Abstract

**Objectives:**

Transitioning into palliative care is psychologically demanding for people with advanced cancer, and there is a need for acceptable and effective interventions to support this. We aimed to develop and pilot test a brief Acceptance and Commitment Therapy (ACT) based intervention to improve quality of life and distress.

**Methods:**

Our mixed-method design included: (i) quantitative effectiveness testing using Single Case Experimental Design (SCED), (ii) qualitative interviews with participants, and (iii) focus groups with hospice staff. The five-session, in-person intervention was delivered to 10 participants; five completed at least 80%.

**Results:**

At baseline, participants reported poor quality of life but low distress. Most experienced substantial physical health deterioration during the study. SCED analysis methods did not show conclusively significant effects, but there was some indication that outcome improvement followed changes in expected intervention processes variables. Quantitative and qualitative data together demonstrates acceptability, perceived effectiveness and safety of the intervention. Qualitative interviews and focus groups were also used to gain feedback on intervention content and to make design recommendations to maximise success of later feasibility trials.

**Conclusions:**

This study adds to the growing evidence base for ACT in people with advanced cancer. A number of potential intervention mechanisms, for example a distress-buffering hypothesis, are raised by our data and these should be addressed in future research using randomised controlled trial designs. Our methodological recommendations—including recruiting non-cancer diagnoses, and earlier in the treatment trajectory—likely apply more broadly to the delivery of psychological intervention in the palliative care setting.

This study was pre-registered on the Open Science Framework (Ref: 46,033) and retrospectively registered on the ISRCTN registry (Ref: ISRCTN12084782).

## Introduction

Cancer is the second most common cause of death globally, responsible for 9.6 million deaths in 2018 [1]. By 2030, an estimated 4 million people will be living with or beyond cancer in the UK [2], and the number of people requiring palliative care is expected to rise substantially [3].

Palliative care aims to maintain quality of life and ease physical and psychological symptom burden. NICE quality standards for adults approaching end of life includes holistic care, encompassing psychological, emotional, and social support [4]. Despite this, people with advanced cancer experience significant psychological and social distress [5, 6]. Transition points, such as that into palliative care, can be particularly psychologically demanding as patients adjust to altered life expectations [7, 8]. This can impact their ability to plan for the future and willingness to engage in advance care planning.

Acceptance and Commitment Therapy (ACT) is a psychological intervention that may support cancer patients approaching end of life. ACT aims not to change or reduce distressing thoughts, but to coach ways of limiting the influence of those thoughts and feelings on day-to-day living and goal achievement by increasing psychological flexibility [9]. ACT acknowledges that distress and suffering are normal, rational reactions to challenging life events. Techniques such as mindfulness, acceptance, and values identification help people to direct their behaviours towards living in the present moment rather than focusing on fears or anxieties. ACT can support people to cope with feelings of grief and demoralisation which may improve quality of life and sense of meaning [10].

ACT has a growing evidence-base in cancer [11], with acceptability indicated in those with advanced disease [12]. In one trial comparing ACT and cognitive behavioural therapy in patients with advanced ovarian cancer, ACT was associated with improved quality of life and reduced distress [13]. In more recent pilot studies, ACT was (non-significantly) associated with decreased symptom interference [14], and significantly improved sleep, distress and hyperarousal [15]. UK-based research reports that it is feasible to deliver ACT to people with advanced cancer, but concluded that more work is needed to robustly test efficacy [16].

Our objectives were to develop and test acceptability and potential efficacy of a brief ACT-based coaching intervention to support people with an incurable cancer diagnosis, at the point of referral to a hospice service. As a secondary aim, our study modelled psychological mechanisms (i.e. psychological flexibility) as a vital first step in fully evaluating ACT as a complex intervention for palliative care.

## Method

The design, including intervention development, has been reported previously [17]. All study protocols received ethical and research governance approvals from the UK Health Research Authority (Ref: 18/WA/0087). The study was pre-registered on the Open Science Framework (Ref: 46,033; Date Registered 12/06/2018) and retrospectively registered on the ISRCTN registry (Ref: ISRCTN12084782; Date Registered 31/01/2021). Study methods were carried out in accordance with principles for medical research involving human subjects as laid down in the World Medical Association Declaration of Helsinki.

### Design

A mixed-methods design was used, as is recommended for trial development [18]. Patient and stakeholder engagement featured heavily to maximise acceptability and implementation [19]. To assess initial effectiveness, we used a single-case experimental design (SCED) [20], allowing for highly controlled delivery alongside patient-centred, in-depth, analysis [21]. SCEDs are experimental designs where the efficacy of an intervention can be closely examined, with each participant providing their own ‘control’ data by completing comprehensive measurement prior to, during, and after implementation of the intervention. Frequent, repeated measurement of process and outcome lend themselves to a mode of within-participant analysis that can be conducted on a very small number of participants. SCEDs are common in psychological intervention research and have established utility in cancer research [22, 23]. Though not designed as a feasibility trial, the design permitted piloting recruitment, intervention and data collection methods.

### Participants

Anyone over 16 years of age at the point of referral into specialist hospice care at recruitment sites (one in England; two in Scotland) following an incurable cancer diagnosis was eligible. Recruitment took place between May 2018 and March 2019. Study invitation took place during the participant’s initial appointment with the hospice specialist nurse.

High attrition was expected given prognosis [24]; accordingly, we excluded those with less than four months life expectancy to maximise trial completion likelihood. We aimed to recruit 20 people, expecting a 50% intervention completion rate. Given that SCED is an idiographic, within-participants approach, this is sufficient for our study aims [25, 26].

### Intervention

The Brief Engagement and Acceptance Coaching for Hospice Settings (BEACHeS) Intervention contained five in-person, one-to-one sessions, each lasting 40–60 min (Table 1). Following an initial assessment, ACT content was delivered over three subsequent sessions, approximately one week apart. The fifth session, one month later, consolidated and maintained gains, and problem-solved difficulties. In-person work was supplemented with home-practice and exercises. Written session summaries were provided to support change and encourage sharing of content with significant others.

**Table 1:** Outline of intervention content

Session	Purpose	Content
1	Module A: Assessment & Engagement	Warmth, empathy, positive regard. History taking, typical responses to transition, beginning baseline monitoring and introducing measurement protocol and concepts
2–4	Module B: Workability^a^	Review of typical responses to distress/suffering and greater contact with the consequences, linking ineffective strategies with control, avoidance and cognitive fusion
	Module C: Awareness	Teaching awareness skills, linking to greater behavioural choice, mindfulness exercises, 5 senses experience, mindful eating a raisin, 10-min mindfulness audio exercise given for homework
	Module D: Openness	Demonstrating the greater effectiveness of willingness to have difficult thoughts and feelings and at the same time, stepping back from such inner experiences
	Module E: Engagement	Linking behavioural effectiveness with desired outcomes and qualities of actions, in order to live with purpose and meaning
5	Module F: Follow-up	Review of progress, barriers to practice, anticipation of future challenges and how ACT skills could be used, behavioural rehearsal of effective responses, commitments to next steps. Ending contact

^a^ The workability module was delivered to all participants at the start of session 2, however, the modular format allowed for awareness, openness and engagement to be delivered in whichever order was most appropriate for each participant [19]

In developing the manual we selected exercises and metaphors that were suitable for participants with a non-curative cancer diagnosis. For example, we avoided those exercise and metaphors common to other ACT intervention manuals that involve the participant considering their *future* life choices, instead developing tailored metaphors more appropriate to those who may be close to the end of life (see our published protocol for more detail) [17]. The manual was peer-reviewed by five independent ACT or palliative care experts.

The intervention was designed to be delivered by appropriately trained Clinical Psychologists or BABCP accredited psychological therapists to enable optimal experimental control, and to establish manual safety prior to delivery by other healthcare professionals in future trials. Two intervention facilitators were appointed and their competency independently assessed against published criteria [27] using video-simulation. Supervision was provided by a Clinical Psychologist and delivery audio-recorded for fidelity checking.

By developing an intervention that is both brief and manualised, we aimed to optimise long term cost-effectiveness and implementation possibilities.

### Procedure and outcome assessments

Eligibility screening was undertaken by hospice-based community specialist nurses who provided study information and gained verbal consent to refer patients to the research team. A researcher then contacted participants by telephone to arrange the initial assessment session, where informed consent was taken and baseline measures assessed. These measures were repeated prior to each subsequent intervention session:

#### Primary outcome: quality of life

Administration of the Functional Assessment of Chronic Illness Therapy (Palliative Care) [28] provided primary outcome data. This scale comprises five quality of life domains—physical (7 items), social/family (7 items), emotional (6 items), functional (7 items) and additional concerns (19 items)—each scored on a scale of 0 (anchored to poorer quality of life) to 4 (anchored to better quality of life). Participants answer based on their experiences over the previous seven days.

#### Secondary outcomes: distress

Distress was assessed using the single-item Distress Thermometer [29], a commonly used screening tool in cancer settings (score range 0 to 10; higher ratings represent worse distress). Participants indicate their distress level over the previous seven days.

#### Postulated Intervention mechanism: psychological flexibility

Psychological flexibility was assessed using the 23-item Comprehensive Assessment of Acceptance & Commitment Therapy (CompACT) [30]; separate component scores—openness to experience, behavioural awareness and valued action—map closely to the three core intervention sessions making this ideal for our study. Subscale scores range from 0 to 42, with higher scores representing greater psychological flexibility.

#### Daily assessments

Daily recordings of key process and outcome measures are integral to SCED studies and were assessed using either a smart-phone based app (PACO: Personal Analytics Companion) or paper-and-pen alternative. Participants completed the Brief Acceptance Measure (BAM) [31], a three-item measure of psychological flexibility (score range 0–30 where high scores indicate greater flexibility), and a single quality of life question where participants indicated their current overall health from 0 (worst imaginable) to 100 (best imaginable). All daily assessments ask participants to provide a rating based on how they were feeling on that particular day.

#### Qualitative data

Participants who completed the intervention, or actively withdrew, were invited to a qualitative interview. A choice of telephone or in-person interview (at the hospice) was offered. The interview schedule aimed to elucidate experiences of study participation, acceptability and perceived intervention effectiveness. Debrief information was provided on study completion.

After closing recruitment, staff involved at each sites were invited to a focus group discussion about challenges and barriers to recruiting participants and supporting the study in the hospice setting. We also sought opinions about different study designs for follow-on research. Interviews and focus groups were audio-recorded and transcribed verbatim for analysis.

### Analysis

In this paper we focus on participants meeting intervention completion criteria, defined as completion of at least four (80%) intervention sessions. Missing data was not imputed. Consistent with SCED best-practice [32], daily assessment data was analysed using visual analysis and within-case statistical analysis. Visual analysis was performed using the SCED Package for R [33]. This uses ordinary least squares regression to create trend lines and Median Absolute Deviation to provide a standardized visualization of data spread, which are considered an advancement on other visual analysis strategies [34, 35]. Aligned with the idiographic nature of SCED research, we refer to participants using pseudonyms throughout our results.

Tau-U tests determined statistical significance of data overlap between variance envelopes and independence of trend lines between the baseline phase (session 1 though to session 2), the active intervention phase (Session 2 through to one week after Session 4), and follow-up (up until to session 5), whilst controlling for baseline trend [26]. Due to the small sample, weekly assessment data was analysed using descriptive methods only.

Participant interview data was analysed thematically [36]. Focus group data was analysed using a framework approach to allow for a structured approach to address study aims [37].

## Results

**Fig. 1:**
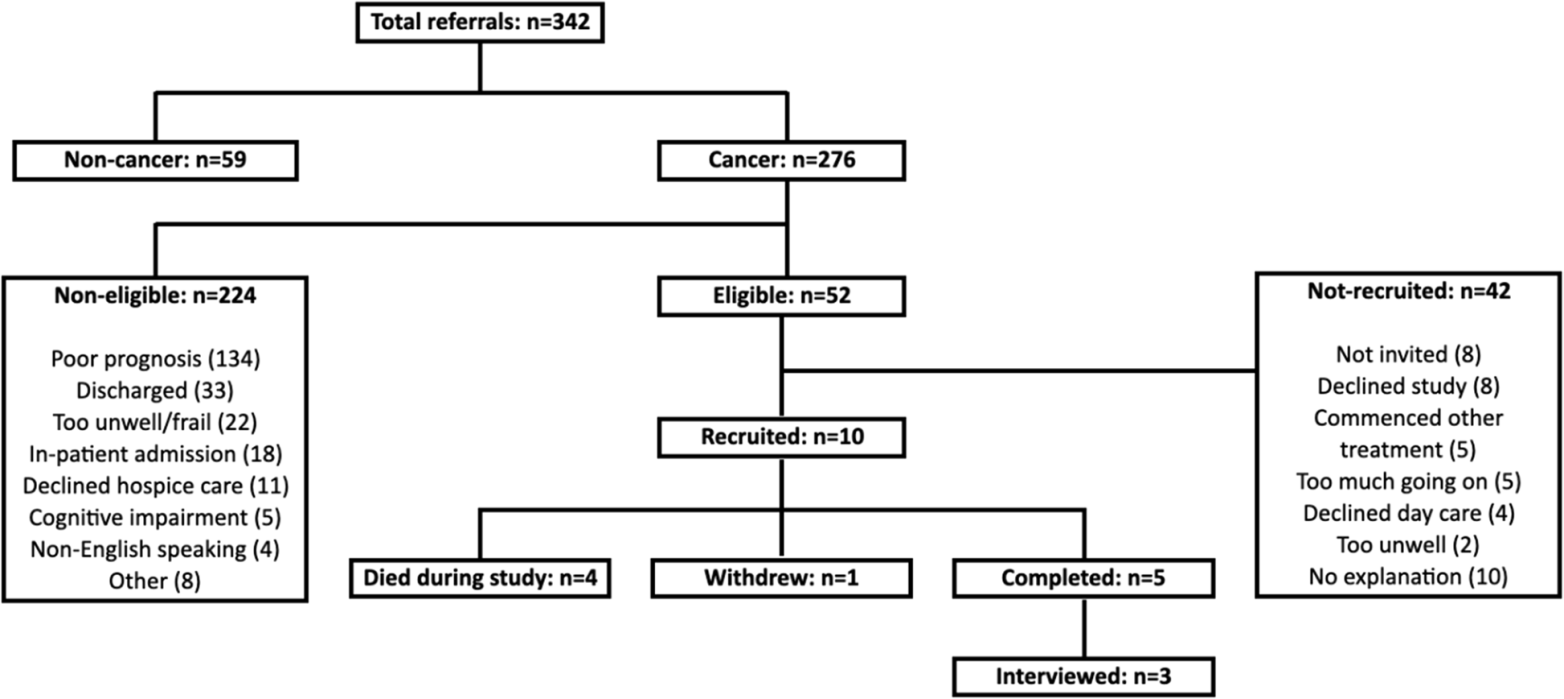
Recruitment, eligibility and attrition

Recruitment took longer than anticipated and after nine months only 10 participants had consented. Only 15.2% of hospice referrals met eligibility criteria, and just 19.2% of those consented (Fig. 1).

The five participants (50%) who completed the intervention provided a sufficient sample size for SCED analysis, in which successful delivery to three participants is considered a minimum to demonstrate reliability of effect [38]. Outcome measure completion was high: no participants missed data on weekly measures, half completed 97% or more daily assessment points (two non-completers failed to engage with daily assessments entirely). Only one participant opted for smart-phone based daily data collection.

Of the participants who did not complete the intervention, one withdrew, and four died part-way through (Table 2).

**Table 2:** Participant (pseudonym) characteristics and intervention engagement

Participant	Clinical description	Engagement / session order
**Intervention completers**		
Elizabeth	58 year old single woman, with breast cancer. Elizabeth moved in with her sister during treatment, fearful of loss of independence and mobility. Her distress levels were low when she entered the intervention. At the time of referral she was undergoing palliative chemotherapy. She accessed orthopaedic services to discuss surgical options for her symptoms. Clinical data indicated that after a good chemotherapy response, Elizabeth was discharged from hospice community services 10 months after referral	Session 1: Module A Session 2: Module B & C Session 3: Module D (3 week gap) Session 4: Module E Session 5: Module F Interview: 7 weeks later
James	81 year old widower, with oesophageal cancer. James had grown children living locally, with grandchildren. He was receiving palliative radiotherapy. James was moderately distressed, reviewing life meaning. He continued to have active engagement with a variety of hospice support services	Session 1: Module A Session 2: Module B & D Session 3: Module E Session 4: Module C Session 5: Module F Interview 13 weeks later
Graham	66 year old with oesophageal cancer and chronic obstructive pulmonary disorder. Graham was living with a supportive partner, and had children in other parts of the UK. He was not especially distressed but low in mood at times. Graham continued to access hospice day services	Session 1: Module A Session 2: Module B & C Session 3: Module D Session 4: Module E Session 5: Module F Interview 2 weeks later
Andrew	73 year old man with prostate cancer. He had a supportive wife and grown children. Andrew stopped conventional treatment when he was referred to hospice care. Overwhelmed by his diagnosis, and distressed, he accessed mainly emotional and psychological support. Other services used included occupational therapy	Session 1: Module A Session 2: Module B & E Session 3: Module D Session 4: Module C Withdrew 3 weeks later
Michelle	46 year old woman with cervical cancer. She had a long history of interpersonal difficulties, relatively chaotic lifestyle, and previous episodes of psychological problems which were now stable. Michelle had completed palliative chemotherapy and sought emotional and benefits advice. She appeared avoidant of thinking of her illness	Session 1: Module A Session 2: Module B & C Session 3: Module D Session 4: Module E Session 5: Module F (Over an extended 13 week period)
**Withdrawn or deceased participants**		
Sally	48 year old woman with lung cancer. Sally had children and young grandchildren, and was supported by her husband. At the time of entering the intervention, she was moderately distressed. Sally was receiving only palliative treatment and died 4 months after referral to the hospice. She had received visits from the hospice nursing service, but did not access any other hospice support or care services	Session 1: Module A Session 2: Module B & C Formally withdrew from study 11 weeks after this, and died two weeks later
Mary	73 year old woman, with pancreatic cancer. She had a long history of psychological difficulties, although her mental health was currently stable. Mary was living alone, supported by her daughter. She appeared resilient throughout her time in the study. She was being cared for with ongoing pain and symptom management and in addition to hospice nurse visits, she accessed benefit support and diabetic nurse care. Mary died almost three months after her referral into hospice services	Session 1: Module A Session 2: Module B & C Withdrew from study the following week, and died five weeks later
John	71 year old man with bladder cancer, who was undergoing palliative chemotherapy. He had increasing levels of pain and nausea and became socially withdrawn when told his cancer wasn’t curable. After two sessions he withdrew from the study because he decided to return to work. John was admitted to hospital five months after referral into hospice community services. He died in hospital a few weeks later	Session 1: Module A Session 2: Module B & C Withdrew from study five weeks later
Daniel	69 year old man, with cancer of the digestive organs and peritoneum. Daniel was married, with grown children, and was well supported by his wife. Daniel became increasingly unwell over a short period time following hospitalisation for a suspected infection. He was receiving palliative treatment for pain, fatigue and agitation. Daniel died at home seven weeks after referral to hospice community services	Session 1: Module A Withdrew from the study before Session 2
Michael	72 year old man, diagnosed with colon cancer and liver metastases. Michael was living with his wife, and with children and grandchildren. Michael was relatively accepting, with low levels of distress throughout the intervention. He was being treated primarily for pain and accessed physiotherapy services through the hospices. Michael was admitted to inpatient care at the hospice following a hospital stay. He died in the hospice three months after his first referral	Sessions 1, 2 and 3 covered only Module A, and took place over a longer-than-specified period of time (7 weeks): patient died two weeks later

Compared to previous literature, our sample reported poorer baseline quality of life [28]. Intervention completers were slightly below threshold for clinically-significant distress (< 4) at baseline [39]. Physical and functional quality of life were lower in non-completers indicating poorer baseline health status (Table 3).

**Table 3:** Descriptive data for outcome and psychological flexibility at baseline (full sample)

		**Full sample**		**Intervention completers (*n* = 5)**		**Non-completers (*n* = 4) **^**d**^	
	Possible score range	Mean	Standard deviation	Mean	Standard deviation	Mean	Standard deviation
Quality of life^a^							
Physical	0 – 28	13.48	6.32	15.2	7.36	11.75	5.06
Social	0 – 28	20.44	5.19	19.68	5.57	21.2	5.37
Emotional	0 – 24	15.35	6.47	13.2	7.01	17.5	5.68
Functional	0 – 28	11.91	5.86	13.06	7.71	10.75	2.87
Palliative specific	0 – 76	59.58	19.70	59.9	16.46	59.25	25.95
Distress^b^	0 – 10	4.42	2.40	3.6	3.05	5.25	0.96
Psychological Flexibility^c^	0 – 126	83.83	26.42	84.4	35.64	83.25	12.91

^a^FACIT-PAL sub-scales (higher scores indicate better quality of life)

^b^Higher score indicates more distress

^c^Higher score indicates more psychological flexibility

^d^No data available for one participant (Daniel)

### Intervention effectiveness

**Fig. 2:**
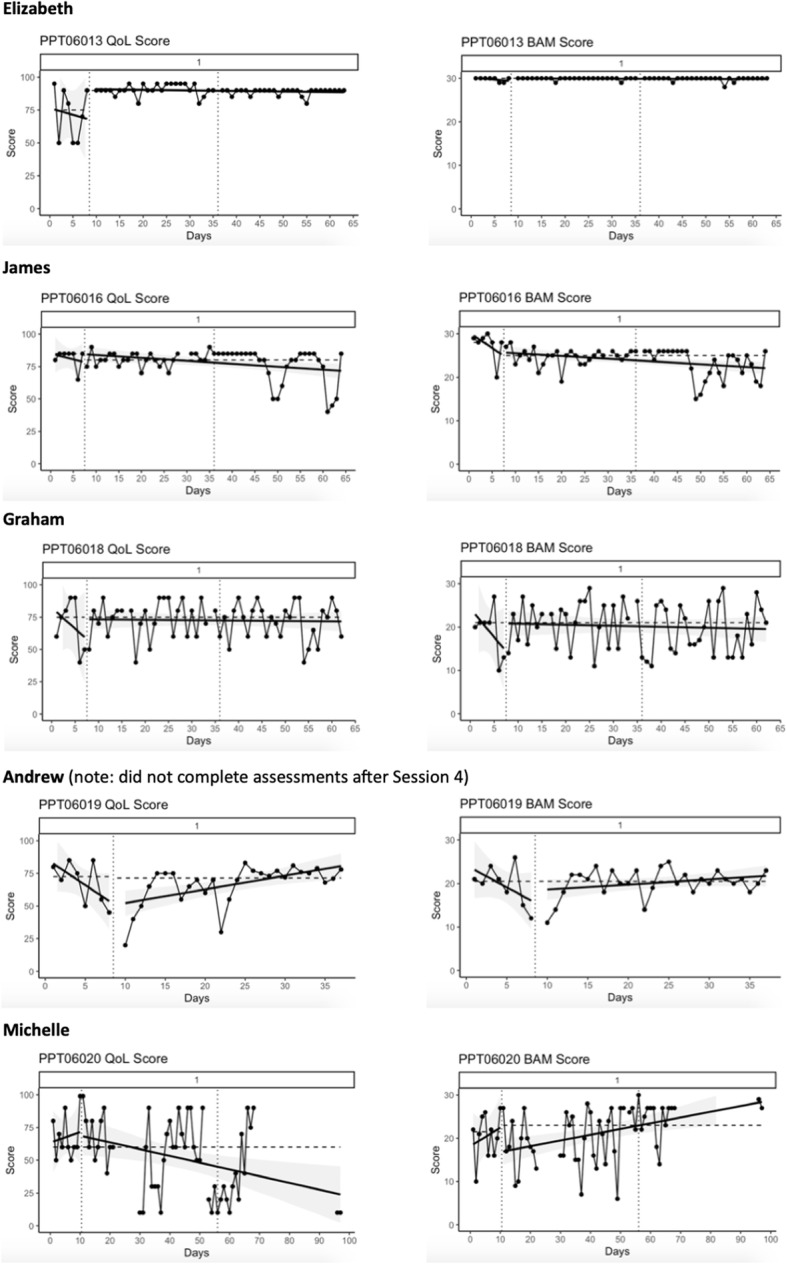
Graphical summary of daily assessed, single-item quality of life question (left) and psychological flexibility assessed using the BAM (right). Ordinary least square regression trend lines are displayed with the solid straight line; Median Absolute Deviation variance is indicated by the shaded area behind the data plots); dotted vertical lines indicate end of baseline phase and end of intervention phase

Visual analysis highlights problematic ceiling and floor effects in daily BAM and single-item QoL assessments (Fig. 2). Additionally, baseline periods lacked ideal levels of stability, and there was little distinction between experimental phases (baseline to intervention and intervention to follow-up). Weekly assessments offered more assessment sensitivity and variability over time.

Elizabeth, Andrew, and Graham all showed deteriorating quality of life during baseline; for both Elizabeth and Andrew this then stabilized and improved during the intervention. Tau-U analysis (Table 4) confirmed this improvement was significant in only one case (Elizabeth). The lack of further significant change from intervention to follow-up indicates that gains were maintained. James and Michelle reported non-significant decreasing quality of life through the intervention.

**Table 4:** Tau-U statistical analysis of changes in daily assessed quality of life (single-item) and psychological flexibility (BAM)

	**Quality of Life**		**Psychological flexibility**	
	**Baseline to intervention**	**Intervention to follow-up**	**Baseline to intervention**	**Intervention to follow-up**
Elizabeth	Tau-U = 0.62 *p* = .01*	Tau-U = -0.26 *p* = .11	Tau-U = .23 *p* = .35	Tau-U = -.09 *p* = .60
James	Tau-U = -0.11 *p* = .64	Tau-U = -.31 *p* = .06	Tau-U = -0.71 *P* < .01**	Tau-U = -.43 *p* = < .01**
Graham	Tau-U = -.05 *p* = .86	Tau-U = -.05 *p* = .076	Tau-U = .10 *p* = .70	Tau-U = -.064 *p* = .69
Andrew	Tau-U = .29 *p* = .16	Missing data	Tau-U = .29 *p* = .16	Missing data
Michelle	Tau-U = -.32 *p* = .09	Tau-U = -.35 *p* = .06	Tau-U = -.308 *p* = .69	Tau-U = .56 *p* < .01*

^*^indicates a statistically significant change between intervention phase in the desired direction

^**^indicates a statistically significant change contrary to expected direction of effect

Elizabeth’s data demonstrates a ceiling effect on the BAM. Both James and Graham reported decreasing psychological flexibility, and in James’s case this was significant throughout. Andrew reported a non-significant increase in psychological flexibility from baseline to intervention, and Michelle demonstrated significant improvement in psychological flexibility occurring between intervention and follow-up.

**Fig. 3:**
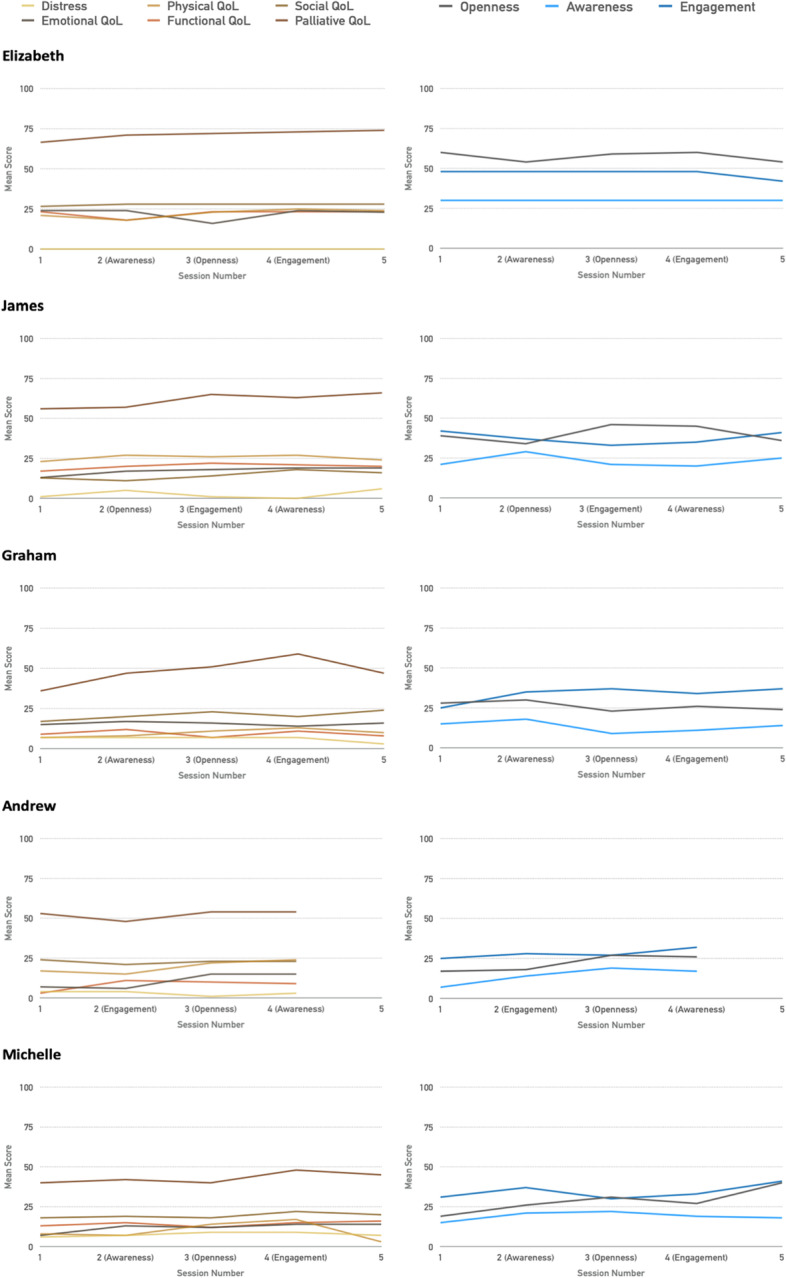
Graphical summary of weekly assessed outcomes (left) and sub-components of psychological flexibility (right) with module topic specified per participant

Weekly assessment change scores (Fig. 3) demonstrate stability in distress for four participants, and improvement for the fifth; for some (e.g. Elizabeth) there was a measurement ceiling-effect which prevented improvement being recorded. Four showed improvement in at least some quality of life sub-domains, however two participants (Graham, Michelle) demonstrate a considerable decrease through follow-up. For Michelle, more so than Graham, it is encouraging that distress and psychosocial quality of life stayed stable, even though physical health deteriorated.

Two participants (James, Andrew) demonstrated a clear mapping of improvement of sub-components of psychological flexibility (openness, awareness, engagement) following delivery of those specific modules, and this is consistent with the pattern of outcome improvements too.

The sample is too small for confident conclusions to be drawn, but there is some evidence that increased psychological flexibility maps closely onto buffering distress increases and maintaining psychosocial quality of life, even where physical and functional quality of life deteriorated.

### Qualitative feedback

Three intervention completers took part in interviews, and fifteen staff took part in focus groups. These qualitative data are described in three themes: intervention effectiveness, intervention acceptability, and suggestions for future research.

#### Perceived intervention effectiveness

All participants reported benefits from taking part, including facing the reality of end of life:

*“…I wasn’t sure whether I would die with dignity…he helped me through that.” (James)*

And being able to discuss fears and emotions honestly:

*“…you can sit and talk and you’re not holding anything back…somebody different to your family…” (Graham)*

Participants describe using the coping skills taught outside of the intervention sessions:

*“…it was the meditation part of it. Yeah. Mhhm because I used to do that quite a lot… And it was yeah, it was really good…the other night I used it to get to sleep.” (Elizabeth).*

The intervention helped participants to accept their current circumstances:

*“I always had this mental picture of lying on my death bed surrounded by my family and I realised that that wasn’t going to happen, you know. And he worked me through my feelings with that one.” (James).*

Staff involved in focus groups reported positive perceptions too, based on their conversations with participants:

*“…he only made it to two sessions, two or three, and he had a great benefit…his wife noticed a difference as well…she felt that he was definitely…lighter when he came back from session.” (FG2)*

#### Intervention acceptability

Patients found the intervention acceptable, with participants responding positively about intervention content. Intervention length was thought appropriate by all but one participant (who expressed a preference for a shorter intervention); they warned that expecting more would have been difficult due to the challenge of deteriorating health:

*“I found them just the right…I don’t think I could have went any longer.” (Graham)*

There was some variation in opinions around preferred metaphors:

*“…it’s just not the way that I think…other people it might be, but not me.” (Elizabeth)*

And some participants suggested that the intervention might benefit more people if the language was simplified.

*“I don’t know whether I was rationalising it, I don’t know, but I put my difficulty down to age. Comprehension, you know, trying to comprehend…I think a lot of people would get benefit if it was just tempered down a wee bit.” (James).*

Patients were supportive of delivering the intervention in the hospice setting:

*“…certainly much more convenient, it’s a conducive environment as well…it’s nice that you can come to this protected environment…” (James)*

And their views on offering the intervention at this specific transition point into palliative care were also positive:

*“I would say it would be the best time. Cause people can get into a mindset if you leave it too long and they’ll never get out of it. So if you get them at the beginning…get there quick.” (Elizabeth)*

Reference here to ‘the beginning’ might also support a move to earlier recruitment in the care pathway, given that the boundary between curative and palliative care is also moving earlier.

Staff focus groups added further depth to understanding acceptability issues. Staff reported that some participants struggled with practical components, having expected more traditional ‘talk’ therapy. One participant told staff that time between sessions was essential to think through answers and reflections on exercises:

*“…he’s [the patient] extremely intelligent…these questions that were asked made him have to do homework and have to think more, and he found them extremely challenging.” (FG3).*

Staff talked frequently about the prevalence of distress and the need for psychological interventions, highlighting a perceived expectation (from patients) that this will be supported by hospice nurses.

*“…sometimes you go and patients are quite anxious…you’re non-threatening…there’s a bit more of an intimacy there because you’re in their house…you’ve build that little bit of rapport…” (FG3).*

However, some staff thought that public perceptions of hospices as only having relevance for end of life might be problematic:

*“the people who are most likely to turn down coming to the hospice …[are] the people who would actually benefit the most…” (FG2).*

#### Future research using the BEACHeS intervention

Participants were asked to reflect on how the BEACHeS intervention might evolve in the future, and there was a range of views expressed. Support for re-working this intervention for group delivery was not strong: participants felt this would have prevented in-depth discussion, and willingness to openly discuss the difficult emotions that accompany a terminal diagnosis. One participant described being ‘open’ to the idea and could see a potential benefit, but for another it would have been a strong deterrent.

*“…culturally, we’re not ready for this dynamic type of group work. People are guarded…you know, my illness is personal to me…No, I think the one-to-one is much more therapeutic…” (James)*

Views on the inclusion of caregivers in the intervention were varied, but there was not a strong appetite for their inclusion:

*“It’s just my partner, I dinnae ken if she’d want to come in or no eh. She’s funny that way isn’t she?” (Graham)*

There were mixed views from patients on whether the intervention should be nurse-led, with some feeling that psychologists were the experts:

*“ I think leave the palliative care staff to do the palliative care; let’s do the drug regime and let’s make you comfortable at night and let’s get you food, and leave that side of it, but leave the grey-matter stuff to the psychologists.” (James)*

Importantly, staff felt that recruiting at hospice referral did not adequately capture patients at the point of psychological transition, but that this occurred earlier in the cancer trajectory:

*“…often the patients that we’ve got have already gone through that stage.” (FG2)*

The proposed solution was to recruit from the hospital setting:

*“the erm hospital CNS’s you know working with oncology and seeing cause they see them at a much earlier stage, they might be the people that help a bit earlier.” (FG2)*

Sufficiently trained, these staff expressed interest in delivering this new intervention:

*“…I think it would fit in with what we do if we had that proper training…” (FG2).*

## Discussion

We aimed to develop a manualised ACT-based coaching intervention for people with non-curative cancer transitioning into palliative care. We explored acceptability and initial effectiveness for improving quality of life and distress.

With relatively minor modifications, we were able to use existing ACT metaphors and exercises to ensure suitability for palliative care populations. Participants’ qualitative data indicated intervention acceptability, and our 50% completion rate is in line with other research [15]. Participants reported that they learned useful emotion management techniques and appreciated the space to talk about worries and fears. Some individual preferences against specific metaphors and exercises emphasises the need for alternative content options, though the broad use of metaphor was acceptable.

Though designed for weekly delivery, this was not practicable; in the context of rapid and unpredictable health status changes, flexibility is essential. For some, this may mean a less rigid delivery schedule over a greater number of weeks; for others a more intensive delivery over a shorter period of time. Flexibility around setting may also be beneficial to reduce burden of travel or resistance to visiting the hospice setting. Given the growth of telehealth over the past year, it may also be useful to consider virtual delivery via telephone or video conferencing.

Our sample had poorer baseline quality of life than comparative samples [28]. Still, weekly assessments demonstrated preliminary evidence for a positive effect on this outcome. Distress remained stable for most: as a tentative hypothesis, we believe our intervention may have buffered against the increased distress often associated with physical health deterioration [5]. Our expected increase in psychological flexibility was not demonstrated, but this may be because of inadequate follow-up duration.

Daily assessment data failed to robustly demonstrate the statistically significant Tau-U effects that we would hope from SCED studies. We believe this may, in part at least, be due to measurement floor and ceiling effects. The complexity of palliative care[40] might mean that daily assessments are over-sensitive and traditional approaches of recording outcomes as a calculated ‘average’ over a set-period of time might be more psychometrically informative.

### Strengths and limitations

Our intervention was designed to have long-term cost-effectiveness through brevity and manualisation, comparing favourably against other ACT trials in palliative care [13, 16]. Rather than progressing straight to a feasibility trial, we developed the intervention in an empirical and evidence-based way using integrated mixed-methods. Doing this using an established and real-world delivery (SCED) method enabled us to demonstrate safety, acceptability and tentative efficacy. We ensured optimal likelihood of success by assessing the competency of facilitators, and rates of attrition and missing data were all positively indicated.

There are limitations. First, we did not include outcome assessment after the follow-up session. Second, we were unable to recruit partial-completers to the qualitative interview; most died prior to invitation. In future trials, this feedback would be beneficial. Third, as outlined above, we had some issues with our measures. Finally, there was a bias towards recruitment of an already accepting, low-distress sample.

### Implications

SCEDs do not require a control group, but without this it is premature to conclude that outcome improvements are a direct result of our intervention. These data, however, certainly support further testing using designs incorporating randomisation and blinding. Such work should aim to investigate the distress-buffering hypothesis generated from our interpretation of weekly assessments.

Our work highlights important methodological considerations for future psychological intervention research in palliative settings. Given that our recruitment rates were lower than anticipated, future trials may wish to extend recruitment beyond only people with cancer for added generalizability and potential population capture. Despite staff buy-in, 42 otherwise eligible patients were not invited to participate; clinical gatekeeping is an often-reported research barrier [41] which must be overcome.

Our four-month life-expectancy eligibility criteria (a) excluded many hospice referrals, and (b) did not effectively reduce attrition. Recruitment earlier in the treatment pathway is, therefore, recommended, for example by shifting the point of study recruitment to the hospital setting at the point of diagnosis of incurable disease, rather than after a hospice-care referral has been made. Provision at this earlier time point would enable participants to make better and longer use of the skills learnt, particularly before the more unpredictable end of life phase begins, and may offer broader benefit to those who may be reluctant to otherwise engage with hospice services.

For those with stable illness, intervention completion rates were high. However, trial length was problematic for those with deteriorating health. Alternative, compressed, delivery is worthy of investigation, though this raises challenges related to homework and skill practice. Furthermore, those who completed the intervention were typically lower in distress at baseline; future work should recruit a sample with higher distress at baseline in whom higher efficacy might be hypothesised.

Regarding delivery and content, two final points are noteworty. First, whilst our intervention is reasonably pitched for adults, changes to presentation and content might be needed for younger populations. Second, our use of highly-trained psychological therapists is infeasible for long-term implementation: future research should explore whether fidelity and efficacy are maintained in delivery by other members of the healthcare team.

## Conclusion

Our data adds to the growing evidence base supporting the use of ACT for people with advanced cancer. We successfully developed an acceptable intervention, and demonstrated some level of initial effectiveness. We have demonstrated the utility (and challenges) of using SCEDs in this setting, notwithstanding some measurement issues. We are planning a feasibility trial of this intervention with an adapted design to improve recruitment and attrition rates.

## Data Availability

The datasets generated and/or analysed during the current study are not publicly available in accordance with our informed consent agreement with participants, but are available from the corresponding author on reasonable request in a de-identified format.
